# Empagliflozin-Induced Nondiabetic Ketoacidosis in Advanced Alcoholic Liver Disease

**DOI:** 10.14309/crj.0000000000001787

**Published:** 2025-08-04

**Authors:** Sudheer Dhoop, Mohammed Shehada, Ahmad Abdelrahman, Abdel-Rhman Mohamed, Thomas Sodeman

**Affiliations:** 1 Division of General Internal Medicine, University of Toledo College of Medicine and Life Sciences, Toledo, Ohio; 2 Division of Gastroenterology and Hepatology, University of Toledo College of Medicine and Life Sciences, Toledo, Ohio

**Keywords:** empagliflozin, nondiabetic ketoacidosis, advanced alcoholic liver disease, heart failure

## Abstract

A 67-year-old man with advanced alcoholic-associated liver disease developed nondiabetic ketoacidosis shortly after starting empagliflozin for congestive heart failure. He presented with high anion-gap metabolic acidosis, elevated beta-hydroxybutyrate levels, and hypoglycemia. The condition resolved promptly after empagliflozin discontinuation and initiation of intravenous fluids, thiamine, and dextrose therapy. This case highlights the risk of early-onset ketoacidosis in patients with cirrhosis and alcohol use treated with sodium-glucose cotransporter-2 inhibitors, emphasizing careful patient selection and vigilant early monitoring of electrolytes and renal function. As the application of sodium-glucose cotransporter-2 inhibitors expands into managing refractory ascites in cirrhotic patients, further studies are required to confirm their safety.

## INTRODUCTION

Sodium-glucose cotransporter-2 inhibitors (SGLT2Is) act as weak diuretics, inhibiting glucose and sodium reabsorption in the renal proximal tubules.^1^ They increase the risk of diabetic ketoacidosis, particularly euglycemic diabetic ketoacidosis, as reduced serum glucose promotes ketogenesis.^2^ While SGLT2Is show potential for managing refractory ascites in cirrhosis,^3,4^ safety data in advanced liver disease is limited.^5,6^ We report a case of nondiabetic ketoacidosis that occurred shortly after empagliflozin initiation in a patient with advanced alcoholic-associated liver disease.

## CASE PRESENTATION

A 67-year-old man presented to the emergency department for symptoms of dyspnea and progressive bilateral lower extremity edema. Pertinent history included primary hypertension, hyperlipidemia, heart failure with improved ejection fraction with implantable cardioverter defibrillator for primary prevention, and nicotine use disorder. He also notably had decompensated alcohol-associated liver disease attributed to prior alcohol use disorder and congestive hepatopathy complicated by ascites and underwent paracentesis yielding 3.4 L with serum-albumin-ascites gradient of 1.8 g/dL and total ascitic protein of 2.3 g/dL 1 year prior. In addition, he had portal hypertensive gastropathy seen on screening esophagogastroduodenoscopy shortly afterward. The patient was hemodynamically stable but required oxygen via nasal cannula. Laboratory findings revealed mild, chronic hyponatremia (134 mEq/L), thrombocytopenia (95 × 10^3^/µL), but were otherwise unremarkable (Table 1). Alanine aminotransferase was 38 U/L, aspartate aminotransferase was 42 U/L, and total bilirubin was 1.2 mg/dL.

**Table 1.** Electrolyte trends for patient before, during, and after ketoacidosis presentation

Day	Context	Sodium	Potassium	Magnesium	Chloride/Bicarbonate	BUN/Cr	Glucose	Alb	T. Bili	INR	pH	MELD-3.0	Child-Pugh
1	HF admission	134	4.0	1.9	102/26	12/0.98	87	2.9	1.2	1.7	7.36^a^	15	8
5	Discharge with SGLT2I	136	3.8	—	89/29	14/1.04	89	—	—	—	—	—	—
9	Readmission	140	3.1	1.5	98/12	18/1.20	69	—	—	—	7.21^a^	—	—
11	Discharge	138	4.9	2.2	100/22	20/1.24	102	—	—	—	—	—	—
20	Clinic discharge Follow-up	134	3.8	—	108/26	10/0.63	72	3.1	1.4	1.5	—	14	7

Abbreviations: Alb, albumin; BUN, blood urea nitrogen; Cr, creatinine; HF, heart failure; SGLT2I, sodium-glucose cotransporter-2 inhibitor.

^a^ The pH is from venous blood gas sample.

He was found to have pulmonary edema on chest X-ray and was admitted for congestive heart failure (CHF) exacerbation. Symptoms improved with IV furosemide, but echocardiography revealed an ejection fraction of 35%, right ventricular failure, and dilated cardiomyopathy. After 4 days of inpatient diuresis, oxygen needs resolved, and his home regimen of spironolactone 50 mg/day, furosemide 40 mg/day, and valsartan 80 mg/day he reported compliance with were continued. Empagliflozin 10 mg/day was added at discharge as guideline-directed CHF therapy.

Four days postdischarge, the patient re-presented with severe nausea, abdominal pain, and generalized fatigue ongoing for the past 24 hours. Laboratory findings revealed hypokalemia (3.1 mEq/L), hypomagnesemia (1.5 mg/dL), high anion-gap metabolic acidosis (anion gap of 28, bicarbonate of 12 mmol/L), and hypoglycemia (69 mg/dL). Venous blood gas demonstrated acidemia (pH 7.23), with beta-hydroxybutyrate markedly elevated at 8.2 mmol/L and a lactate of 1.5 mmol/L. Hemoglobin A1c was notably normal at 4.9%, and serum ethanol levels were undetectable. Imaging revealed mild hepatomegaly, nodular liver indicative of cirrhosis, and persistent small-volume ascites without acute pathology.

On this presentation, the patient reported unchanged, chronic alcohol intake (1–2 12-ounce beers daily). He denied any changes in appetite, eating 2 carbohydrate-dense meals per day. There were no documented episodes of alcohol intoxication, withdrawal, or alcohol-associated hepatitis. The patient's empagliflozin was stopped, and he was given intravenous fluid with thiamine and dextrose at a rate of 50 mL/hours for 48 hours with prompt resolution of acidosis and symptoms. He presented asymptomatic to his cardiologist's office for a general follow-up 2 weeks after discharge where laboratory work revealed normal electrolytes and renal function (Table 1).

## DISCUSSION

Our report describes a unique case of empagliflozin-associated nondiabetic ketoacidosis in a patient with advanced alcohol-associated liver disease. Prior reports involve similar patients with T2DM developing euglycemic diabetic ketoacidosis after SGLT2I therapy initiation.^7^ Unlike these cases, our patient was nondiabetic and presented with features more consistent with alcoholic or starvation ketoacidosis—hypoglycemia, markedly elevated beta-hydroxybutyrate levels, and rapid clinical improvement upon thiamine and dextrose-based fluids.^8^ Given the rapid symptom onset, stable alcohol and food intake, and no new medications, we attributed the ketoacidosis to empagliflozin.

Our patient's CHF was attributable to dilated cardiomyopathy, which is often associated with alcohol use disorder.^9^ Alcohol metabolism generates ketone bodies and patients with cirrhosis even abstaining from alcohol have higher ketone body generation from physiological stress than those without cirrhosis.^10^ Therefore, alcohol use status and liver disease should be additional considerations before initiating SGLT2Is. Although serum ethanol was undetectable at presentation, there was no phosphatidylethanol test ordered, which can detect alcohol consumption in the past 2-4 weeks and stratify extent of use.^11^ If further cases like ours emerge, phosphatidylethanol before SGLT2I initiation may be warranted. Nonetheless, our patient reported daily alcohol consumption, which, in the context of advanced liver disease, may have had disproportionate metabolic effects and likely contributed to the development of ketoacidosis. Notably, when the empagliflozin was initiated, the patient's serum creatinine was 0.4 mg/dL higher than his clinic baseline although it was marked as normal by the electronic health record. Serum creatinine is often lower in cirrhosis secondary to sarcopenia and acute kidney injuries may be missed.^12^ This may have been an indicator of a prerenal acute kidney injury at discharge, which may have predisposed the patient to ketoacidosis. Therefore, careful patient selection and monitoring of postinitiation electrolytes and renal function are paramount as our case and prior literature demonstrates that ketoacidosis can occur as early as 3-4 days after SGLT2I initiation in patients with advanced alcohol-associated liver disease.^7^

Our case provides cautionary insight on SGLT2Is use in advanced liver disease. Of trials where SGLT2Is were initiated in cirrhosis, including alcoholic-associated cirrhosis, there was no incidence of ketoacidosis.^3,4,13,14^ These studies, however, did not include those with active alcohol use like in our case. More real-world data to assess for risk of ketoacidosis with SGLT2Is in liver cirrhosis are needed. This question will become increasingly relevant as SGLT2Is may have a role in refractory ascites management^3–6^ and have been shown to be beneficial in Metabolic-Associated Steatotic Liver Disease,^15^ a rising etiology of cirrhosis.^16^ Until further safety data are available, caution should be advised before starting SGTL2Is in patients with advanced liver disease even with moderate alcohol use.

## DISCLOSURES

Author contributions: S. Dhoop: conception, literature review, manuscript writing, and editing; M. Shehada: manuscript writing and editing; A. Abdelrahman: manuscript editing; A. Mohamed^:^ manuscript editing; T. Sodeman: review and supervision. S. Dhoop is guarantor of the article.

Financial disclosure: None to report.

Informed consent was obtained for this case report.

## ABBREVIATIONS:

µL: microliter
dL: deciliters
g: gram
L: liters
mEq: miliequivalents
mg: milligrams
mmol: millimole
SGLT2Is: Sodium-glucose cotransporter-2 inhibitors
U: units
